# Circulating Levels of Glial Cell Line-Derived Neurotrophic Factor (GDNF) in Schizophrenia: a systematic review and meta-analysis

**DOI:** 10.1186/s12888-025-06498-9

**Published:** 2025-01-29

**Authors:** Omran Davarinejad, Saeid Komasi, Mohammad-Taher Moradi, Farzaneh Golmohammadi, Maryam Bahrami, Hamed Esmaeil Lashgarian, Masumeh Jalalvand, Sara Hookari, Fatemeh Kazemisafa

**Affiliations:** 1 Clinical Research Development Center, Imam Khomeini and Mohammad Kermanshahi and Farabi Hospitals, Kermanshah University of Medical Sciences, Kermanshah, Iran; 2 Department of Neuroscience and Psychopathology Research, Mind GPS Institute, Kermanshah, Iran; 3 Sleep Disorders Research Center, Health Policy and Promotion Institute, Kermanshah University of Medical Sciences, Kermanshah, Iran; 4 Department of Medical Biotechnology, Faculty of Medicine, Lorestan University of Medical Sciences, Khorramabad, Iran

**Keywords:** Glial cell line-derived neurotrophic factor, GDNF, Meta-analysis, Schizophrenia

## Abstract

**Background:**

Glial cell line-derived neurotrophic factor (GDNF) has emerged as a potential biomarker for schizophrenia (SCZ). However, GDNF levels remain unclear in affected individuals compared to healthy controls. Therefore, we aimed to calculate a pooled estimate of GDNF levels in patients with SCZ in comparison with healthy controls.

**Methods:**

A systematic search was performed in PubMed, Scopus, Web of Science, and Science Direct for published studies from the first date available up to 17 June 2024. Twelve studies (*n* = 817 patients and 691 healthy controls) were included in the meta-analysis. Subgroup analyses and meta-regression were performed, addressing heterogeneity and publication bias.

**Results:**

Random-effects estimates (*d* = –0.80, *p* < 0.001) of the present meta-analysis revealed a significant mean difference in GDNF levels between SCZ patients and healthy controls. Subgroup analyses indicated that the standardized mean difference of GDNF was larger in European samples (*d* = –1.01, *p* ≤ 0.001) than in the Asian population (*d* = –0.61, *p* = 0.011). Non-medicated SCZ patients (*d* = –1.08, *p* ≤ 0.001) exhibited lower GDNF levels than those on medication (*d* = – 0.70, *p* = 0.004). Additionally, patients with a disease duration of ≥ 10 years showed lower levels of GDNF (*d* = –0.93, *p* = 0.058 versus* d* = –0.82, *p* = 0.002).

**Conclusions:**

The findings suggested that GDNF may be a promising biomarker and therapeutic target for schizophrenia. Future research should focus on elucidating the mechanisms underlying altered GDNF levels and exploring its implications for treatment strategies.

**Supplementary Information:**

The online version contains supplementary material available at 10.1186/s12888-025-06498-9.

## Background

Schizophrenia (SCZ) is one of the most prevalent functional psychiatric disorder, characterized by a range of symptoms including hallucinations, slurred speech, delusions, and impaired cognitive function [1]. Approximately 20 million individuals worldwide are affected by this disorder, with prevalence rates increasing significantly by 65.85% from 1990 to 2019 [2, 3]. Despite decades of research, the underlying causes of SCZ remain largely elusive, complicating the establishment of definitive diagnostic criteria and a comprehensive understanding of its pathophysiology. These challenges have hindered efforts to develop effective treatments [4].

Risk factors for SCZ are generally classified into genetic, environmental, psychosocial, developmental, and biological domains [5]. Among these biological factors, neurotrophic factors—particularly glial cell line-derived neurotrophic factor (GDNF)—are critical for elucidating the pathophysiology of SCZ [6–12].

GDNF plays a crucial role in the nervous system by promoting the survival, differentiation, and function of dopaminergic neurons, which are essential for normal brain activity [13]. It primarily exerts its effects through interactions with the GDNF family receptor-α1, leading to the activation of Ret tyrosine kinase receptors. This signaling cascade is vital for several key processes, including neuronal survival, neurite outgrowth, and synaptogenesis—each of which contributes to the maintenance of healthy neural networks [14].

In addition to its role in neurodevelopment, GDNF has demonstrated significant neuroprotective effects in various experimental models. For instance, it helps preserve neuron-glial networks under hypoxic conditions and regulates the expression of hypoxia-inducible factor 1-alpha (HIF-1α) [15]. The neuroprotective actions of GDNF are mediated through several intracellular pathways, notably the RET kinase receptor complex and the PI3K/Akt signaling pathway [16–18]. These pathways are critical for supporting neuronal health and functionality by promoting cell survival and mitigating cellular stress.

Several studies have reported associations between GDNF levels and various mental disorders, including mood disorders [19], anxiety [20], depression [21], and bipolar disorder [22]. Notably, alterations in GDNF levels have been observed in patients with major depressive disorder [21] and bipolar disorder [22], suggesting a potential role for GDNF in the pathophysiology of these conditions. However, the role of GDNF as a biomarker in psychiatric disorders—particularly SCZ—remains a topic of ongoing debate. While GDNF is crucial for the survival and function of dopaminergic neurons [13], its precise role in the pathophysiology of SCZ has yet to be fully elucidated.

Research on serum levels of GDNF in schizophrenia reveals several critical gaps. While some studies indicate lower GDNF levels in patients with SCZ [6–12, 23, 24], others report no significant differences or higher levels [25–27]. These heterogeneous results highlight the need for larger standardized studies. Additionally, associations between GDNF and clinical symptoms remain preliminary and require validation across diverse populations.

Furthermore, the biological mechanisms linking GDNF to schizophrenia are poorly understood. There is also insufficient exploration of how comorbid conditions may influence GDNF levels. Our current understanding is limited to original studies that do not adequately address differences between sociodemographic subgroups or consider how sample size and study quality impact findings.

Addressing these gaps could enhance our understanding of GDNF's role in schizophrenia and improve diagnostic and therapeutic approaches. The primary aim of this meta-analysis is to calculate a pooled estimate of circulating levels of GDNF in patients with SCZ compared to healthy controls. By synthesizing data from existing studies, we seek to clarify the inconsistencies in reported GDNF levels and their potential associations with clinical symptoms. To achieve our secondary objectives, we conducted subgroup and meta-regression analyses to assess the impact of several sociodemographic and clinical factors—including geographic region, sex ratio, age of illness onset, disease duration, daily dose of Chlorpromazine, and total score of the Positive and Negative Syndrome Scale (PANSS), study year, and study quality-on GDNF levels. This comprehensive approach aims to enhance our understanding of GDNF's role in schizophrenia and its potential as a biomarker for diagnosis and treatment.

## Methods

### Search strategy and databases

This study conducted a systematic search across PubMed, Web of Science, Scopus, and ScienceDirect. Our systematic review and meta-analysis were carried out following the methodological steps outlined in the Preferred Reporting Items for Systematic Reviews and Meta-Analyses (PRISMA) guidelines, registered in PROSPERO by code CRD42024576318 and at Kermanshah University of Medical Sciences, Kermanshah, Iran (ethical approval number: IR.KUMS.REC.1403.401). Two authors (F.K. and M.M.) independently searched from the first date available up to 17 June 2024. We used the key search terms according to the PICOS formula [28]: ((schizophrenia[Title/Abstract]) OR (schizophrenia[MeSH Terms])) AND (((GDNF[Title/Abstract]) OR (glial cell line-derived neurotrophic factor[MeSH Terms])) OR (glial cell line-derived neurotrophic factor[Title/Abstract])) in PubMed, obtaining 51 articles. We also used the key search terms: (TITLE-ABS-KEY (GDNF) OR TITLE-ABS-KEY ("glial cell line-derived neurotrophic factor") AND TITLE-ABS-KEY (schizophrenia)) in Scopus, obtaining 171 articles. Additionally, we searched for (glial cell line-derived neurotrophic factor" or GDNF and schizophrenia) in Science Direct, obtaining 16 articles. We used the search terms: ((TS = (GDNF)) OR TS = ("glial cell line-derived neurotrophic factor ")) AND TS = (schizophrenia) in the Web of Science, obtaining 81 articles.

### Selection criteria

The inclusion criteria were as follows: 1) patients with SCZ, 2) healthy control subjects without current mental disorder, 3) a reported GDNF levels in serum, 4) adults 18 years of age or older, regardless of gender, 5) case–control studies, and 6) clinical trials and cohort studies if reporting baseline data. The studies were excluded based on the following criteria: 1) animal or in vitro studies, 2) reviews or meta-analyses except for manual search of their references, 3) studies focused on genetic polymorphism or gene expression, 4) studies lacking a control group of healthy individuals, and 5) abstracts without full text.

### Screening of studies

**Fig. 1:**
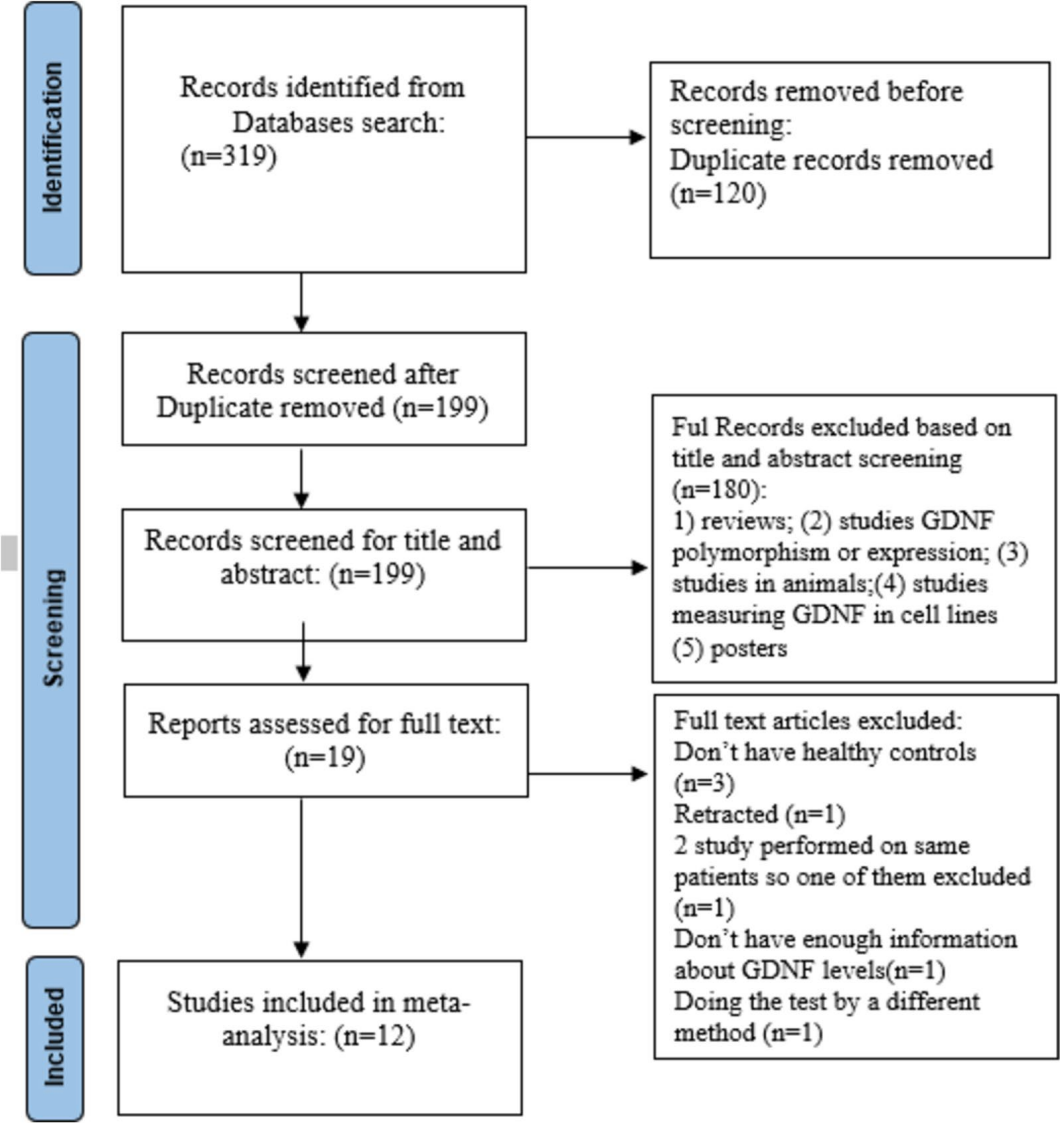
PRISMA flow diagram of the study procedure

Data were extracted by two independent researchers (F.K. and M.M.). After extraction, both researchers compared their data and, in the case of any discrepancies, collaborated to reach a consensus to ensure that the extracted data were accurate and valid. All the search results were imported into EndNote v20. After eliminating duplicates, two authors independently reviewed the titles, abstracts, and full texts of the potentially eligible articles. In cases of disagreement between the two authors, the third author (O.D.) was consulted to resolve the matter. A total of 319 studies were identified through electronic searches. After eliminating duplicates, 199 articles were retained. A review of the titles and abstracts of these articles resulted in the exclusion of 180 articles. Consequently, 20 articles were selected for full-text evaluation. Following this evaluation, 8 articles were excluded, resulting in a total of 12 articles published between 2014 and 2023 being included in the meta-analysis. Figure 1 illustrates the PRISMA flow diagram.

### Extraction of data

The variables extracted from the studies included: 1) mean and standard deviation of the GDNF levels for each group, 2) the number of patients and controls, 3) age and gender of patients and controls, 4) scores in PANSS scales in patients, 5) the mean dose of Chlorpromazine equivalents used by patients (mg), 6) duration of treatment in patient group, 7) characteristics of measurement (ELISA kit utilized), 8) study design (case–control, cohort and cross-sectional), and 9) age of onset of illness. When a study measured GDNF blood levels at two different time points [7, 9, 24, 27], we used the GDNF levels at the baseline period. In some studies that report the median GDNF levels [12, 23], we calculated the mean and standard deviation of the GDNF levels using other parameters explained by researchers [29]. In two studies, SCZ patients were categorized into two groups, with GDNF values reported separately for each group. The combined mean was then utilized to derive the final values for both groups [6, 24].

### Evaluation of quality

The methodological quality of the studies was evaluated using the Newcastle–Ottawa Scale (NOS) specifically designed for case–control studies, which assesses the risk of bias in observational research. This scale consists of 8 items divided into three key components: the selection of cases and controls, the comparability of the groups, and the ascertainment of exposure. Each of these components was rated using the star system to provide a comprehensive assessment. Each study can receive a score between 0–9, and a score above 6 is considered acceptable [30]. All twelve studies received a score of 6 or higher and were thus included in the meta-analysis.

### Statistical analysis

A meta-analysis was conducted to determine the pooled effect size of the standardized mean difference (Cohen's *d*) of the GDNF between cases with SCZ and healthy controls. The studies were aggregated based on sample size, mean, and standard deviation of GDNF levels in both cases and controls. Pooled effect sizes for the between-group differences are presented along with 95% confidence intervals (95% CI) in a forest plot. Because all individual studies were from Asian and European regions, we also calculated pooled estimates for the geographic subgroups, the results of which were depicted by another forest plot. We analyzed several other subgroups as follows: age of onset of the disease (< 25 years vs. ≥ 25 years), the average length of the disease duration (< 10 years vs. ≥ 10 years), Chlorpromazine daily dose (< 500 mg vs. ≥ 500 mg), and the average total score of PANSS (scores < 80 vs. ≥ 80). We used several meta-regressions with the method of moments to assess the effect of mean age of samples, sex ratio, quality of the study, year of study, age of illness onset, disease duration, Chlorpromazine dose, and the PANSS scores on the pooled estimates. An *I*^2^ of 50% and above was used to detect heterogeneity and, in this case, the random-effects method was used to compute all pooled estimates. However, we also reported the estimates for the fixed-effects method. Egger's statistic was employed to detect potential publication bias, which was depicted using a funnel plot. All hypotheses were tested at a significance level of *p* < 0.05, utilizing the Comprehensive Meta-Analysis (CMA.2) software.

## Results

### Study and sample characteristics

This meta-analysis includes data from 1,510 participants, including 817 individuals diagnosed with SCZ and 691 healthy controls. The included studies were published between 2014 and 2023 and exhibited a range of sample sizes varying from 48 to 282 participants. The mean age of participants ranged from 24.4 ± 5.7 years to 52.85 ± 6.4 years. In many studies, the control group was paired with the case group based on gender and age. The antipsychotic dosage ranged from 100 to 748 mg of chlorpromazine equivalents per day. The characteristics and significant findings from the studies included in the analysis are outlined in Table 1.

**Table 1:** Characteristics of the included studies in the meta-analysis

NOS	Sample	statistical analysis	Mean Total PANSS	Measurement methods GDNF levels	Disease duration	Gender (M/F)	Age (M ± SD)	Healthy control (*n*)	SCZ (*n*)	*N*	Country	Study and year
6	Serum	ANOVA X 2 test Student’s t-test	-	ELISA	9.1 ± 7.3	51/64	SCZ: 35.9 ± 8.2 Controls: 34.9 ± 7.3	52	63	115	Japan	Niitsua et al., [26]
7	Serum	Student’s t-test Pearson correlation test- Spearman correlation test	109.56	ELISA	4.2 ± 5.4	46/44	SCZ: 32.1 ± 10.2 Controls: 53.0 ± 9.4	45	45	90	Turkey	Tıkır et al., [12]
6	Serum	Chi-square Fisher Exact Tests two-sided Mann–Whitney test	70.28	ELISA	15.1 ± 9.3	Not reported	SCZ: 24.4 ± 5.7 Controls: Not reported	37	37	74	Turkey	Çetin et al., [23]
7	Serum	Student’s t-test- Kolmogorov–Smirnov test- Mann–Whitney U test	84.54	ELISA	Not reported	55/0	SCZ: 37.5 ± 10.7 Controls: 36.3 ± 10.1	43	42	85	turkey	Turkmen et al., [9]
8	Serum	Student’s t-test- Kolmogorov–Smirnov test Pearson’s and Spearman’s correlation -	121.4	ELISA	Not reported	48/0	SCZ: 36.3 ± 9.8 Controls: 36.6 ± 9.0	28	20	48	turkey	Akkus et al., [27]
7	Serum	Chi2 test- one-way ANOVA- Kolmogorov–Smirnov test	74.71	ELISA	11.7 ± 8.5	56/55	SCZ: 36.4 ± 9.9 Controls: 38.2 ± 11.5	78	33	111	Turkey	Tunca et al., [22]
8	Serum	ANOVA- Bonferroni test	-	ELISA	27.0 ± 2.8	149/0	SCZ: 49.7 ± 6.8 Controls: 46.8 ± 10.7	40	109	149	China	Tang et al., [24]
8	Serum	chi‐squared- ANCOVA	56.6	ELISA	25.0 ± 7.1	124/0	SCZ: 52.9 ± 6.4 Controls: 52.5 ± 5.7	53	75	128	China	Feiye et al., [6]
8	Serum	Mann–Whitney U test- ANOVA	75.3	ELISA	24.5 ± 6.0	44/59	SCZ: 25.0 ± 5.9 Controls: 26.5 ± 6.6	50	58	108	China	Xiao et al., [8]
7	Serum	Levene's test- ANOVA- Mann–Whitney U-test	89.58	ELISA	Not reported	21/103	SCZ: 32.4 ± 9.9 Controls: 40.2 ± 14.1	48	55	103	Poland	Skibinskaa et al., [25]
7	Serum	independent sample t-tests- ANOVA- earson's correlation	74.2	ELISA	11.4 ± 7.7	87/70	SCZ: 37.8 ± 11.9 Controls: 40.0 ± 12.5	77	138	215	China	Xiao et al., [7]
6	Serum	Pearson correlation test Student’s t-test-	80	ELISA	6.0 ± 4.0	142/140	SCZ: 25.0 ± 4.0 Controls: 26.0 ± 4.0	140	142	282	China	Pang et al., [11]

*GDNF* Glial cell line-derived neurotrophic factor, *SCZ* Schizophrenia, *NOS* Newcastle–Ottawa Scale**,** *PANSS* Positive and Negative Syndrome Scale

### Differences in GDNF levels

**Fig. 2:**
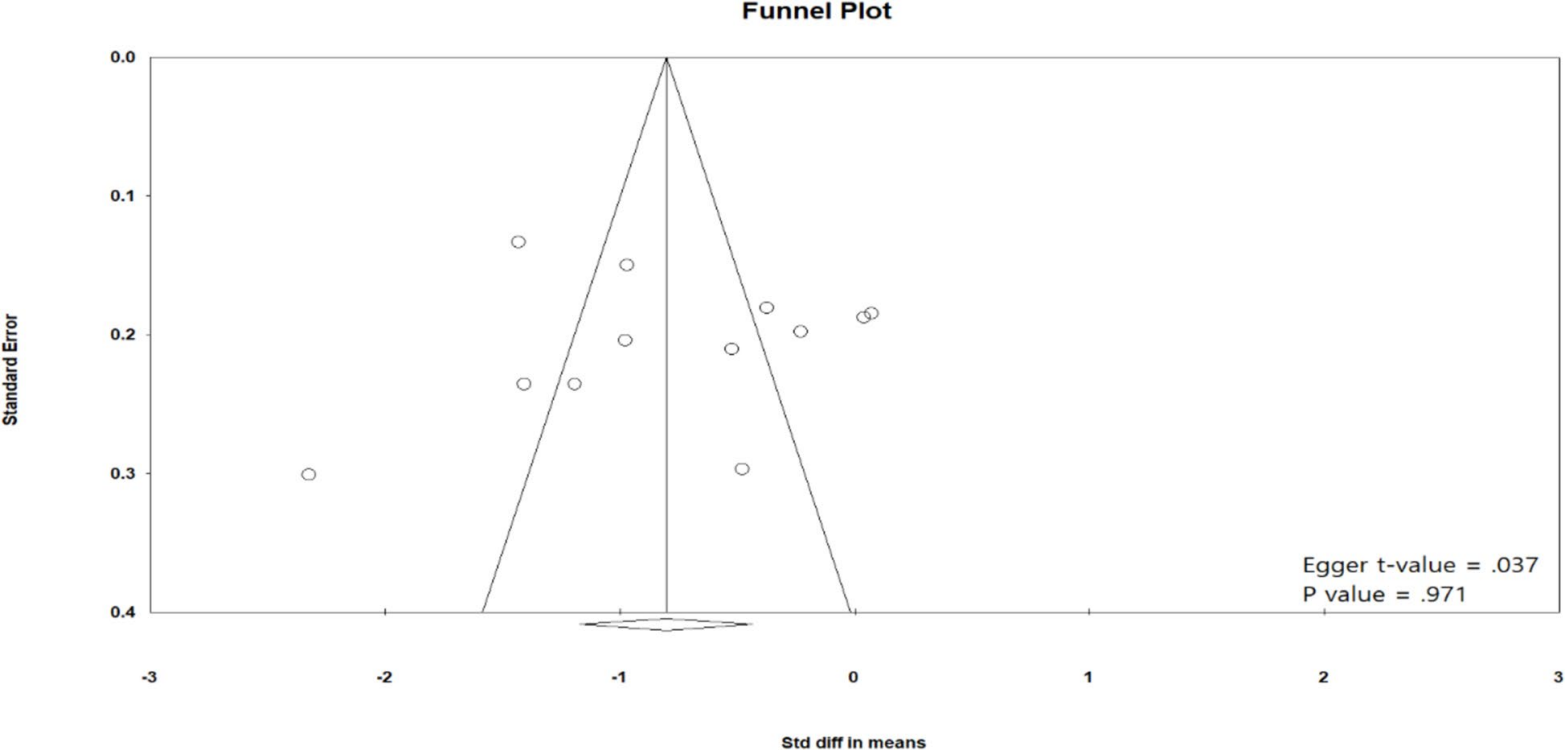
Funnel plot for identifying publication bias

**Fig. 3:**
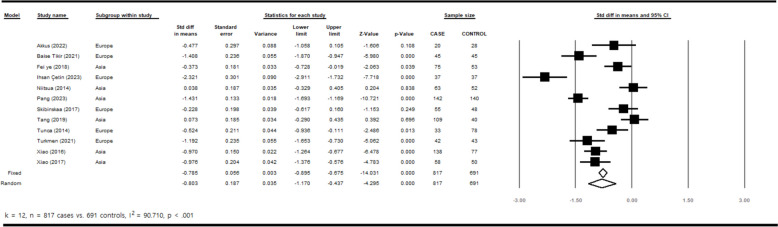
Forest plot for the standardized mean difference in the GDNF between cases and controls

The estimates from the random-effects model are preferable since *I*^*2*^ is significant. The funnel plot (see Fig. 2) did not show any publication bias (Egger *t* value = 0.037, *p* = 0.971). Figure 3 shows the standardized mean difference in the GDNF between cases and controls. As can be seen, there is a significant difference between SCZ and healthy controls in both the random-effects (*d* = –0.80, *p* < 0.001) and the fixed-effects (*d* = –0.79, *z* = –14.031, *p* < 0.001) estimates.

### Meta-regression and subgroup analysis

To find the source of heterogeneity in this study, Meta-regression analysis was conducted for age of onset, duration of the disease, Chlorpromazine dose, total PANSS score, age mean, sex ratio, year of study, and quality of study between the groups, showing an affected *p*-value for sex ratio and year of publication (< 0.05). Table 2 shows the details of the results of subgroup analysis and meta-regression analysis. When we estimated the standardized mean differences for the geographic subgroups (Asian vs. European samples), subgroup analyses indicated that the standardized mean difference of GDNF was larger in European samples. The random-effects estimates were –0.61 [CI: –1.13, –0.10; *p* = 0.019] and –1.01 [CI: –1.59, –0.43; *p* < 0.001] for the Asia and Europe regions, respectively. Other subgroups analyzed included age of disease onset. The results showed relatively equivalent levels of GDNF in both subgroups. The random-effects estimates were –0.77 [CI: –1.45, –0.09; *p* = 0.027] for age of onset < 25 years and –0.67 [CI: –1.25, –0.09; *p* = 0.024] for age of onset ≥ 25 years. When we estimated the standardized mean differences for the average duration of the disease (< 10 years vs. ≥ 10 years), the results showed a significant difference between the subgroups. The random-effects estimates were –0.93 [CI: –1.89, 0.03; *p* = 0.058] for the disease duration < 10 years while it was –0.82 [CI: –1.34, –0.30; *p* = 0.002] for the disease duration ≥ 10 years. In our subgroup analysis of subjects with SCZ, we found that GDNF levels were lower in non-medicated individuals compared to those who were medicated. The random-effects estimates were –1.08 [CI: –1.18, –0.22; *p* = 0.004] in non-medicated patients, while it was –0.73 [CI: –1.33, –0.82; *p* < 0.001] for medicated patients. A significant difference was shown between the subgroups with different doses of chlorpromazine. The random-effects estimates were –0.29 [CI: –1.01, 0.42; *p* = 0.420] for < 500 mg daily dose while it was –0.68 [CI: –1.15, –0.21; *p* = 0.005] for ≥ 500 mg daily dose. The subgroup analysis for the total score of PANSS showed relatively equivalent levels of GDNF in the subgroups. The random-effects estimates were –1.00 [CI: –1.51, –0.48; *p* < 0.001] for total PANSS < 80 and –0.96 [CI: –1.47, –0.44; *p* < 0.001] for total PANSS ≥ 80. Finally, meta-regression analysis showed that the pooled estimates are positively affected by the mean age of the sample (*d* = 0.05, *p* = 0.015) and negatively by the year of study (*d* = –0.14, *p* = 0.006; See Table 2).

**Table 2:** Result of subgroup analysis based on random effect model

Subgroups	*k*	*n*	*I* ^2^	*p*	Std. diff in means	95% CI		*p*	*z*
						Lower	Upper		
Geographic region									
Asia	6	997	92.97	0.001	−0.61	−1.13	−0.1	0.019	−2.34
Europe	6	511	88.95	0.001	−1.01	−1.59	−0.43	0.001	−3.42
Medication									
Yes	9	1095	92.49	0.001	−0.70	–1.18	−0.22	0.019	−2.97
No	3	413	26.41	0.001	–1.08	–1.33	−0.82	0.001	−8.38
Age of onset (meta-regression)					−0.07	−0.29	0.16	0.567	−0.57
< 25 years	5	545	92.45	0.001	−0.77	–1.45	−0.09	0.027	−2.21
≥ 25 years	4	548	90.25	0.001	−0.67	−1.25	−0.09	0.024	−2.26
Duration of the disease (meta-regression)					0.03	−0.02	0.08	0.195	1.29
< 10 years	3	487	95.46	0.001	−0.93	−1.90	0.03	0.058	−1.89
≥ 10 years	6	785	91.00	0.001	−0.82	−1.34	0.23	0.001	−3.6
Chlorpromazine dose (meta-regression)					0.00	0.00	0.00	0.82	0.23
< 500 mg	3	479	92.45	0.001	−0.29	−1.01	0.42	0.42	−0.7
≥ 500 mg	3	324	74.76	0.019	−0.68	−1.15	−0.20	0.001	−3.99
Total PANSS score (meta-regression)					0.00	−0.01	0.02	0.849	0.19
< 80	5	636	88.32	0.001	−1.00	−1.51	−0.48	0.08	−3.8
≥ 80	5	608	88.37	0.001	−0.96	−1.47	−0.44	0.001	−3.65
Meta-regression analysis									
Age mean					0.05	0.01	0.08	0.015	2.43
Sex ratio					0.06	−0.30	0.42	0.738	0.33
Year of Study					−0.14	−0.23	−0.04	0.006	−2.72
Quality of study					0.38	−0.08	0.84	0.105	1.62

### Sensitivity analysis

**Fig. 4:**
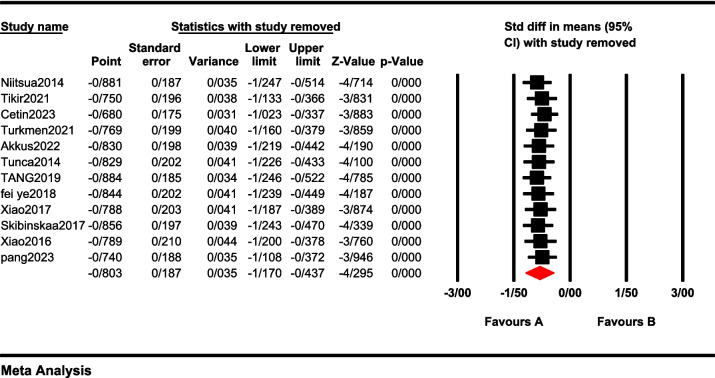
The results of sensitivity analysis

When the pooled effect size indicated significant results, sensitivity analyses were conducted to determine if any individual study was responsible for the notable findings. In this analysis, each study was removed one at a time, and the significance was re-evaluated. No single study accounted for the observed heterogeneity, and the results remained significant in all instances (Fig. 4). In general, we found that heterogeneity remained high in all subgroups and meta-regressions, except when the subgroup was performed in terms of medication status, and non-medicated patients, heterogeneity was ≤ 50% (*I* ^2^ = 26.41*)*.

## Discussion

This meta-analysis, which includes 12 studies with over 1,500 participants, examined the role of GDNF in SCZ. Previous research has established the role of GDNF in various mental disorders, including mood disorders [19, 31], anxiety [20], and bipolar disorder [32]. Our analysis extends these findings to SCZ, confirming an association between lower levels of GDNF and the psychiatric condition. This represents the first comprehensive meta-analysis addressing this subject. The high heterogeneity across studies led us to use a random-effects method for pooled estimates. Since heterogeneity can arise from differences in study populations, interventions, outcomes, or methodologies, we estimated effect sizes across subgroups within studies. Additionally, meta-regression analysis was used to better understand the effects of several moderators on the pooled estimates.

Our findings indicate that patients with SCZ exhibit significantly lower circulating GDNF levels compared to healthy controls. This reduction is particularly pronounced in untreated patients and appears to correlate with the dosage of antipsychotic medication. The majority of evidence supports a negative association between GDNF levels and SCZ, while some studies have reported conflicting results [25–27]. These discrepancies may arise from variations in sample size, patient demographics, and the complex functions of neurotrophic factors in psychiatric conditions.

The meta-regression analysis suggests that combined estimates remain unaffected by variations in the average age of case and control groups, indicating that included studies likely employed age-matching techniques to reduce the impact of confounding variables. Notably, our analysis found higher GDNF levels in studies involving European samples, which may reflect differences in genetics, nutrition, and lifestyle among populations[33–35].

We did not find a relationship between the age of onset and GDNF levels, a finding supported by several studies [7, 10, 12, 23]. This suggests that while GDNF has protective roles for neurons, it may not directly influence the onset of SCZ. Other unknown factors could contribute to disease onset, warranting further investigation.

Additionally, we identified a significant correlation between GDNF levels and disease duration; specifically, longer disease duration is associated with lower GDNF values. This finding aligns with several studies [7, 10] but contradicts others [23, 26], potentially due to small sample size or methodological differences. Furthermore, our study found that GDNF levels were lower in non-medicated subjects compared to those receiving antipsychotic treatment. This highlights the potential impact of these medications on GDNF levels and suggests that antipsychotics may confer neuroprotective effects beyond symptom management by modulating neurotrophic factors.

The relationship between daily antipsychotic dosage and GDNF levels further corroborates the notion that antipsychotics play a significant role not only in managing psychiatric symptoms but also in promoting neuroprotection through the modulation of neurotrophic factors [7]. Given the observed associations between GDNF levels and cognitive function, there is potential for GDNF to serve as a bioecological marker for both diagnosis and treatment response in SCZ.

As a biomarker, GDNF could provide insights into disease progression and therapeutic efficacy. For instance, lower serum GDNF levels might indicate a more severe illness state or poorer treatment responses [7]. Conversely, increases in GDNF levels following antipsychotic treatment could reflect neuroprotective effects and improvements in cognitive function [27]. Therefore, monitoring GDNF levels could enhance personalized treatment approaches for individuals with SCZ.

A study involving mice with a heterozygous GDNF mutation indicated impaired cognitive performance in water-maze tasks [36], underscoring GDNF's role in cognitive function. Additionally, reductions in psychiatric symptoms during antipsychotic treatment was linked to a gradual increase in GDNF levels [7], proposing that GDNF may play a role in both the etiology and pharmacotherapy of SCZ.

Despite our findings supporting a significant association between GDNF levels and SCZ, the relationship between GDNF levels and specific symptom domains remains complex. Our analysis did not identify a direct correlation with total PANSS scores, consistent with findings from two studies [8, 10]. This suggests that while GDNF may influence certain aspects of SCZ, it does not directly correlate with all symptom dimensions assessed by the PANSS. However, some studies have reported correlations between GDNF levels and negative subscales of PANSS [12, 23], indicating a nuanced relationship influenced by variables such as race, treatment type and dosage, and patient age.

### Limitations and future directions

Several limitations should be considered when interpreting our findings. First, the extent to which GDNF can cross the blood–brain barrier remains unclear, limiting our ability to directly link peripheral GDNF levels to central nervous system processes. Second, the limited number of studies and the heterogeneity of patient populations may have influenced the observed effects. Third, most research is cross-sectional, limiting insights into how GDNF levels change over time in relation to treatment response.

Importantly, potential publication bias must also be acknowledged as a limitation. The tendency for studies with positive results to be published more frequently than those with negative or inconclusive findings can skew our understanding of the true relationship between GDNF levels and SCZ. Future research should aim for comprehensive reporting practices that include all results—regardless of outcome—to provide a more accurate picture of this relationship.

Future research should focus on clarifying the precise mechanisms linking GDNF to SCZ, investigating potential therapeutic implications of GDNF modulation, and exploring its role as a biomarker for disease progression and treatment response. Additionally, examining relationships between GDNF and other biomarkers, such as inflammatory markers and neuroimaging findings, may provide further insights into SCZ's pathophysiology.

## Conclusion

This systematic review and meta-analysis underscore the significant association between lower levels of GDNF and SCZ, emphasizing its potential role as a biomarker for this disorder. Our findings suggest that GDNF may not only contribute to SCZ's pathophysiology but also serve as a target for therapeutic intervention, particularly within the context of antipsychotic treatment. While further investigation is warranted regarding the relationship between GDNF levels and various clinical factors such as medication dosage and disease duration, our results advocate for more extensive research into the neuroprotective properties of GDNF. Understanding these dynamics could pave the way for novel treatment strategies aimed at enhancing cognitive function and improving overall patient outcomes in SCZ.

## Supplementary Information

Supplementary Material 1.

## Data Availability

We used publicly available data and no original data was collected.

## Abbreviations

ANOVA: Analysis of Variance
BDNF: Brain-Derived Neurotrophic Factor
CI: Confidence Interval
ELISA: Enzyme-Linked Immunosorbent Assay
GDNF: Glial Cell Line-Derived Neurotrophic Factor
HIF-1α: Hypoxia-Inducible Factor 1-Alpha
NGF: Nerve Growth Factor
NOS: Newcastle–Ottawa Scale
PANSS: Positive and Negative Syndrome Scale
PI3K/Akt: Phosphoinositide 3-Kinase/Protein Kinase B
PRISMA: Preferred Reporting Items for Systematic Reviews and Meta-Analyses
PROSPERO: International Prospective Register of Systematic Reviews
RET: Rearranged During Transfection (a tyrosine kinase receptor)
SCZ: Schizophrenia
