# Supplementary figures and images for: Circulating Levels of Glial Cell Line-Derived Neurotrophic Factor (GDNF) in Schizophrenia: a systematic review and meta-analysis
Source: BMC Psychiatry. 2025 Jan 29;25:83. doi: 10.1186/s12888-025-06498-9 (PMC11780850; doi:10.1186/s12888-025-06498-9)

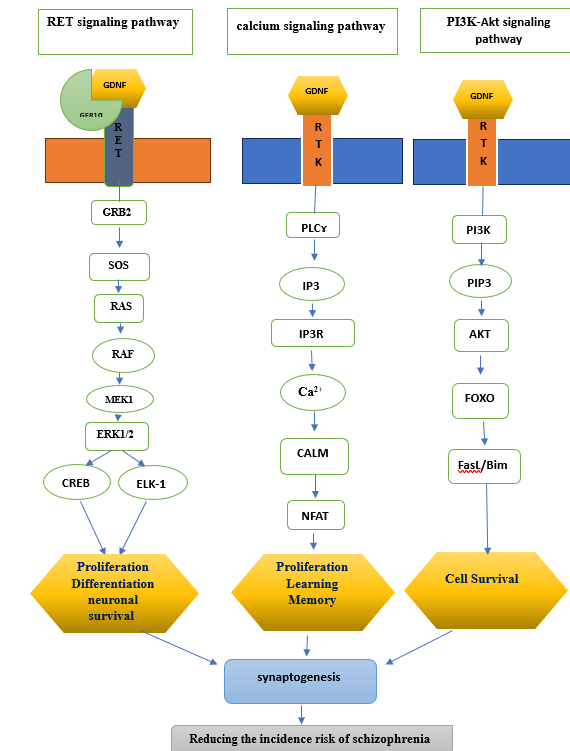

Supplement: Supplementary file 1 — Supplementary Material 1. [file 12888_2025_6498_MOESM1_ESM.png]
